# The Recovery Time between Early Mild Stress and Final Acute Stress Affects Survival Rate, Immunity, Health, and Physiology of Oscar (*Astronotus ocellatus*)

**DOI:** 10.3390/ani13101606

**Published:** 2023-05-11

**Authors:** Mahyar Zare, Elaheh Heidari, Seyedeh Mahsa Hosseini Choupani, Sobhan R. Akhavan, Artur Rombenso, Noah Esmaeili

**Affiliations:** 1 South Bohemian Research Center of Aquaculture and Biodiversity of Hydrocenoses, Faculty of Fisheries and Protection of Waters, Institute of Aquaculture and Protection of Waters, University of South Bohemia in České Budějovice, Na Sádkách, 37005 České Budějovice, Czech Republic; 2 Department of Animal, Marine and Aquatic Biology and Biotechnology, Faculty of Life Sciences and Biotechnology, Shahid Beheshti University, Tehran 1983969411, Iran; 3 Department of Fisheries, Faculty Marine Science, Tarbiat Modares University, Noor 46414-356, Iran; 4 Nelson Marlborough Institute of Technology, 322 Hardy Street, Private Bag 19, Nelson 7010, New Zealand; 5 Agriculture and Food, Livestock & Aquaculture Program, Bribie Island Research Centre, Woorim, QLD 4507, Australia; 6 Institute for Marine and Antarctic Studies, University of Tasmania, Hobart, TAS 7005, Australia

**Keywords:** antioxidant response, blood performance, blood biochemistry, stress physiology, stress response

## Abstract

**Simple Summary:**

Stress plays key roles in the production and the welfare of different aquatic species. Ornamental fish farms systems, such as that of oscar, often experience mild handling stress. However, it is unknown how this early mild stress (EMS) can affect fish and other animals’ growth, physiology, and health. Another question was whether the time of EMS occurrence could affect the fish survival rate to final acute stress. The result indicated that the time of EMS occurrence is a matter, and fish can be recovered from stress if there are no two consecutive stress events.

**Abstract:**

This study investigated how the time interval between the last EMS (netting) and the acute confinement stress (AC stress) at the end of the experiment can influence growth, haematology, blood biochemistry, immunological response, antioxidant system, liver enzymes, and stress response of oscar (*Astronotus ocellatus*; 5.7 ± 0.8 g). Nine experimental treatments were tested, as follows: Control, Stress28 (EMS in weeks two and eight), Stress27 (EMS in weeks two and seven), Stress26 (EMS in weeks two and six), Stress25 (EMS in weeks two and five), Stress24 (EMS in week two and four), Stress23 (EMS in week two and three), Stress78 (EMS in week seven and eight), and Stress67 (EMS in week six and seven). After the nine-week experimental period, while it was not significant, fish exposed to Stress78 (26.78 g) and Stress67 (30.05 g) had the lowest growth rates. After AC stress, fish exposed to Stress78 (63.33%) and Control (60.00%) showed the lowest survival rate. The Stress78 fish displayed low resilience, illustrated by values of blood performance, LDL, total protein, lysozyme, ACH50, immunoglobin, complement component 4, complement component 3, cortisol, superoxide dismutase, catalase, and alanine aminotransferase. In conclusion, gathering consecutive stress and not enough recovery time in the Stress78 group negatively affected stress responsiveness and the health of oscar.

## 1. Introduction

The ornamental fish market size is predicted to be expanded at a compound annual growth rate of 8.5% from 2022 to 2030 and to reach more than USD $11 billion by 2030. The increase in its popularity is driven by decorative purposes in the luxury lifestyle of the new generation, attractiveness, declining stress after a hectic workday, educational purposes, etc. (https://www.grandviewresearch.com/, accessed on 1 March 2023). These features have made this industry one of the most beneficial and efficient agricultural sectors [1]. Intensive production, which is common in ornamental aquaculture, can potentially result in stress [2]. Mild stress, such as netting and confinement stress, is common in aquaculture systems, especially ornamental farming systems, where fish are handled and netted more than edible species due to the regular selling process.

Similar to all vertebrates, the general physiological response of fish to alteration in the homeostasis state is called stress response [3]. Mild stressful conditions can have positive effects, namely, eustress. However, severe stress negatively impacts fish metabolism, such as growth, behaviour, and immunity [3]. The stress response is controlled by two hormonal components (corticosteroids (mainly cortisol) and catecholamines). In the next stage, secondary stress responses (such as glucose and lactate) provide energy sources and oxygen and affect hydromineral imbalance and the immune system [3]. Prolonged exposure of fish to stress can cause adverse effects on homeostasis as stress response costs energy. In other words, metabolically, fish commit energy toward stress responses at the expense of growth. Different fish species and even families and individuals respond differently to stress due to genetic variations [3].

Oscar (*Astronotus ocellatus*) is a popular ornamental fish due to its early maturation, relatively high fecundity, colourful appearance, personality, and cleverness [4]. Limited studies have investigated the adverse effects of stress on this fish species [5,6,7,8,9]. However, studies that tested the effect of early mild stress (EMS) or eustress in aquatic species are limited to our previous studies [4], and other fish studies that lower hypothalamic and brainstem serotonergic responses to stress and cortisol responsiveness were observed [10,11,12]. In our earlier studies, the interaction of EMS with different levels of lipid [4], protein [13], and fish meal replacement [14] were investigated. The highest survival rate after final acute stress (confinement stress) was observed in oscar exposed to two weeks of EMS out of a ten-week trial [4,13]. This output indicated that two weeks of EMS resulted in the highest survival rate after acute confinement stress, regardless of the tested dietary lipid or protein contents. The following hypothesis was raised—whether the timeframe between EMS and final acute stress, or recovery time, can affect the growth, survival rate, and fish health.

To the best of our knowledge, no study comprehensively tested the effect of time between EMS and final acute stress on fish responses in aquatic species, which occurs commonly. Therefore, in follow-up research with the same experimental condition, two times out of ten weeks of scheduled stress, but in different weeks, were considered to investigate fish resilience and these scheduled stress effect on growth performance, haematology, blood biochemistry, immune response, antioxidant activities, stress response, and liver enzymes of juvenile oscar.

## 2. Materials and methods

### 2.1. Animal, Fish Ethic and Experimental Conditions

A national and university Animal Care Committee approved all used experimental protocols (281-1385) [15]. To do this trial, 621 oscar (initial weight: 5.7 ± 0.8 g) were obtained from the Abzian Center (Mahallat, Markazi, Iran). The fish was acclimatised for two weeks before the start of the trial in this farm (this experiment was conducted on this farm) and, during this time, fed with a commercial diet (500 and 150 g/kg crude protein and lipid, respectively). As transportation and handling cause stress, we tried to do these steps as little as possible. Twenty-three fish were randomly distributed into each of twenty-seven rectangular glass tanks (100 L, three aquaria per treatment). Fish were provided with diets three times a day (09:00, 14:00, and 19:00) for ten weeks to ad libitum levels. The photoperiod was set for 12D:12L. To remove faeces and debris, 20–30% of the water in each tank was changed daily with dechlorinated water during the experiment. The water quality parameters were checked throughout the experiment and kept at standard levels, which were explained in our earlier studies from this project [4,13].

### 2.2. Diet Formulation and Experimental Design

The optimum protein, lipid, and fish meal levels and their interaction with EMS have been tested before [4,13,14]. Therefore, an optimum isonitrogenous (450 crude protein/kg feed and 180 g/kg fish meal) and isolipidic (180 g/kg lipid) were used for this study. The diet preparation and formulation processes were reported earlier [4,16]. During the nine-week feeding experiment, two scheduled EMS stress events were conducted in week two and week eight, on both Monday and Friday of that week (Table 1). This involved dragging an aquarium net around the tank for 5 min after a water exchange without actively chasing or removing any fish. The nine applied treatments in the current research were Control, Stress28, Stress27, Stress26, Stress25, Stress24, Stress23, Stress78, and Stress67 (Table 1).

### 2.3. Sample Collection and Growth Performance

Fish were starved for 24 h and anaesthetised using clove oil stock solution (70 ppm) [17]. Standard methods and relationships were used to calculate weight gain, specific growth rate, feed conversion ratio (FCR), daily feed intake, hepatosomatic index (HIS), viscerosomatic index, and condition factor at the end of the experiment [18]. Further, four fish were chosen randomly from each tank, and after collecting blood, their liver and viscera were sampled and weighed.

### 2.4. Chemical Analysis of Diets and Fish Body Composition

The proximate composition of the diet and body samples was measured by AOAC methods [19]. Briefly, crude protein was determined using the Kjeldahl method and an automatic Kjeldahl system (Kjeltec Analyser unit 2300, FOSS, Hilleroed, Denmark). The Soxhlet extraction method was used to examine crude lipids (Soxtec 2050 FOSS FOSS, Effretikon, Switzerland). Moisture was determined by drying samples in a 105 °C oven for 12 h. The ash was determined using a Nabertherm muffle furnace (Model K, Nabertherm GmbH, Lilienthal, Germany) at 550 °C for 4 h.

#### 2.4.1. Blood Collection and Sample Preparation

Four fish from each tank were tested for haematology, immune response, blood biochemistry, antioxidants, and serum enzymes at the end of the experiment (weeks nine and ten). To reduce stress, the fish were anaesthetised with clove oil (50 ppm), and blood samples were quickly collected via caudal vein venipuncture with a sterile 5-mL syringe. Following that, blood was refrigerated for 2 h before serum was collected after centrifuging at 3000× g at 4 °C for 2 min [17] and then stored at –20 °C until further analyses. Then, serum samples were pooled for analysis, and three samples per treatment were considered.

#### 2.4.2. Hematology Profile

Red blood cells (RBCs) and white blood cells (WBCs) were counted in a Neubauer hemocytometer and the Neubauer chamber, respectively, as described before [20]. Further, the haemoglobin (Hb) and hematocrit (Ht) were determined by cyanmethemoglobin and the microhematocrit method [4,21]. Mean corpuscular volume (MCV), mean corpuscular haemoglobin (MCH), mean corpuscular haemoglobin concentration (MCHC) [22], and blood performance (BP) [23] were calculated according to the below formula:

(1) $$\mathrm{Mean}\;\mathrm{corpuscular}\;\mathrm{volume}\;\left(\mathrm{MCV}\right)\left(\mathrm{fl}\right)=\left[\frac{\mathrm{Ht}}{\left(\mathrm{RBC}\;10^{6}/{\mathrm{mm}}^{3}\right)}\right]\times 10$$

(2) $$\mathrm{Mean}\;\mathrm{corpuscular}\;\mathrm{haemoglobin}\;\left(\mathrm{MCH}\right)\left(\mathrm{pg}\right)=\frac{\mathrm{Hb}}{\left(\mathrm{RBC}\;10^{6}/{\mathrm{mm}}^{3}\right)}\times 10$$

(3) $$\mathrm{MCHC}\;=\frac{\mathrm{Hb}}{\mathrm{Ht}}\times 100$$

(4) $$\mathrm{Blood}\;\mathrm{Performance}\;=\mathrm{Ln}\;\mathrm{Hb}\;\left(g/\mathrm{dL}\right)+\mathrm{Ln}\;\mathrm{Ht}\;\left(\%\right)+\mathrm{Ln}\;\mathrm{RBC}\;(\times 10^{5}/{\mathrm{mm}}^{3})+\mathrm{Ln}\;\mathrm{WBC}\;(\times 10^{3}/{\mathrm{mm}}^{3})+\mathrm{Ln}\;\mathrm{total}\;\mathrm{protein}\;\left(g/L\right)$$

#### 2.4.3. Blood Biochemistry, Antioxidant Enzymes Activities, Serum Enzymes and Cortisol

Plasma biochemical parameters, glucose, TP, albumin, globulin, high-density lipoproteins (HDL), low-density lipoproteins (LDL), cholesterol, triglycerides, lactate, alkaline phosphatase (ALP), lactate dehydrogenase (LDH), aspartate transaminase (AST), and alanine aminotransferase (ALT) were analysed using commercial clinical investigation kits (Pars Azmun Kit, Karaj, Iran). The antioxidant enzymes, including superoxide dismutase (SOD), catalase, glutathione peroxidase (GPx), and malondialdehyde (MDA,), were measured using ELISA kits, according to the kit protocol (ZellBio, GmbH, Lonsee, Germany). Cortisol levels in serum were measured using commercial kits (Boditech iCHROMA, Chuncheon-si, Republic of Korea).

### 2.5. Non-Specific Immune Parameters

To determine serum lysozyme, Gram-positive bacteria, sensitive to the lysozyme enzyme method, were used (*Micrococcus lysodeikticus*) [24] as substrates. Alternative complement pathway hemolytic activity (ACH50) was determined by hemolysis of rabbit RBCs (RaABC) [25]. Serum immunoglobulin, complement C3 (C3), and complement C4 (C4) levels were measured by the ELISA method using CUSABIO and MyBioSource kits companies (CUSABIO- CSB-E12045Fh- and CUSABIO, CSB- E09727s) based on the protocol available in the kit packages. The complete methods for measuring these parameters were described in our previous study [26].

### 2.6. Acute Confinement Stress (AC Stress)

After ten weeks of the experiment, oscars exposed to AC stress based on our previous studies to test fish’s ability to tackle stressful situations [4,27]. To do so, after obtaining samples in week 9 (Table 1), ten fish per tank was adjusted with three tanks per treatment. Then, fish rested for one week, and we applied AC stress in week 10. The acute stress was a succession of netting all of the fish in each tank, followed by a 30-s air exposure before being transferred to a plastic mesh bucket at a density of 120 g/L in their original tank for 5 h. Aeration was regularly performed to prevent oxygen depletion and premature death. Following 5 h of stress, blood sampling and serum extraction were performed in the afternoon, as previously described (three fish per tank [4]). The survival rate of fish after 48 h in various treatments is shown in Table 2.

### 2.7. Statistical Analysis

This study used a completely randomised design with nine treatments and three replications. One-way ANOVA and Tukey’s HSD were used to compare treatments. For data with percentages, the transformation was conducted before further analysis. The data (mean ± SDM) were analysed after checking normality and homogeneity of variance. The significant difference in each treatment before and after AC stress was reported according to the independent sample *t*-test (*p* < 0.05). In all analyses, a significant difference between treatments was defined as a difference of 5% or less. SPSS (version 21.0 for Windows) was used to analyse the data. We could not consider the sex effect because we could not realise females and males in this size of fish.

## 3. Results

### 3.1. Growth Performance and Body Composition

In the present research, while it was not significant, the weight gain in Stress78 (26.78 g) was the lowest (Table 2). There was no significant difference in FCR, daily feed intake, HSI, and condition factor, showing that feed efficiency was not affected in this study. Differences in survival rates in the present research were not significant among treatments, suggesting the culture condition was accepted by fish. However, survival after final AC stress in Control (60.0%) and Stress78 (63.3%) was significantly lower than the Stress25 (80.0%), Stress27 (83.3%), and Stress28 (80.0%) (*p* < 0.05). In the present investigation, there was no significant difference among treatments regards protein, fat, ash and moisture contents (Table 3).

### 3.2. Haematology and Blood Biochemistry

**Figure 1:**
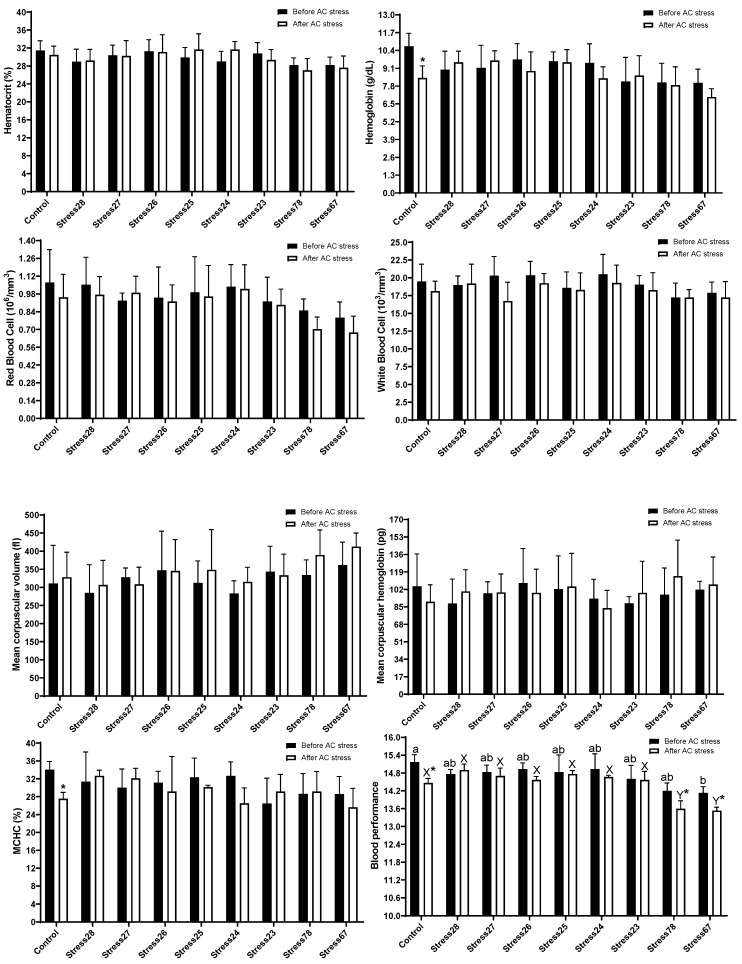
Haematological parameters of oscar exposed to different schedule stress during ten weeks plus data related to after acute confinement stress (AC stress). Values were represented by means ± SDM of triplicate samples. Asterisk indicates the significant difference in each treatment before and after AC stress according to the independent sample *t*-test (*p* < 0.05). The letters a, b and X, and Y indicated significant differences based on the Tukey test among groups before (nine treatments) and after AC stress (nine treatments), respectively.

**Figure 2:**
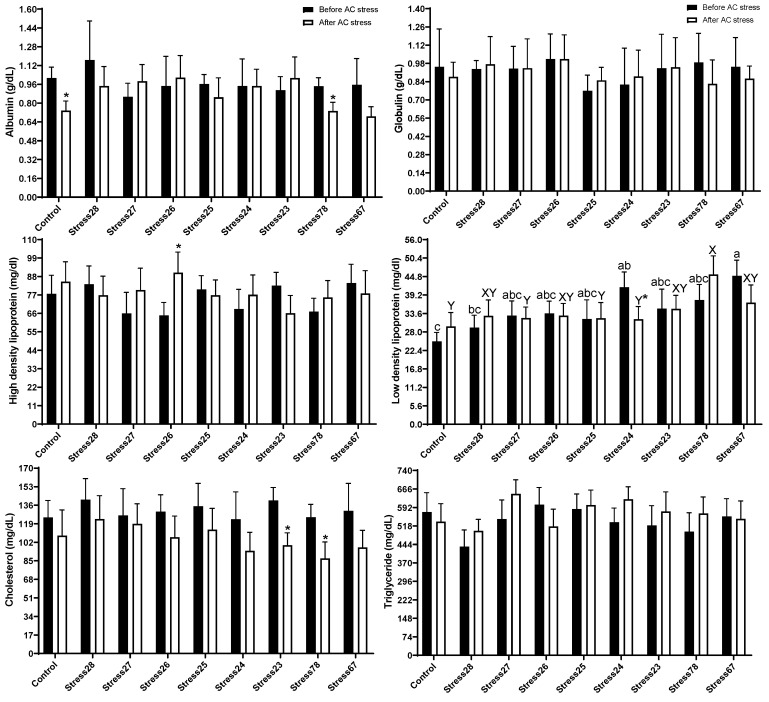
Blood biochemistry parameters of oscar exposed to different schedule stress during ten weeks plus data related to after acute confinement stress (AC stress). Values were represented by means ± SDM of triplicate samples. Asterisk indicates the significant difference in each treatment before and after AC stress according to the independent sample *t*-test (*p* < 0.05). The letters a, b, c and X, and Y indicated significant differences based on the Tukey test among groups before (nine treatments) and after AC stress (nine treatments), respectively.

Figure 1 indicated that there was no significant difference in Ht, Hb, RBC, WBC, MCH, MCV, and MCHC among groups. The AC stress decreased Hb, MCHC, and BP in the Control group compared to before stress and also, BP in Stress78 and Stress67 fish were lower after AC stress. Before stress, the BP had lower content in Stress67 (14.13) group than in the Control (15.18) (*p* < 0.05). After AC stress, oscar experienced the Stress67 (13.53) and Stress78 (13.59) schedules had a lower value of BP than the other treatments (*p* < 0.05). In the current research, blood biochemistry was changed with stress schedules (Figure 2). Before stress, there was no change in albumin, globulin, HDL, triglyceride, and cholesterol levels. Stressed groups, including Stress24 (41.56 mg/dL) and Stress67 (45.03 mg/dL), had higher values of LDL than the Control (25.13 mg/dL). After stress, the same results for LDL value were observed, so that Stress78 (45.46 mg/dL) had a higher value than Control (29.70) (*p* < 0.05).

### 3.3. Immune and Stress Response

**Figure 3:**
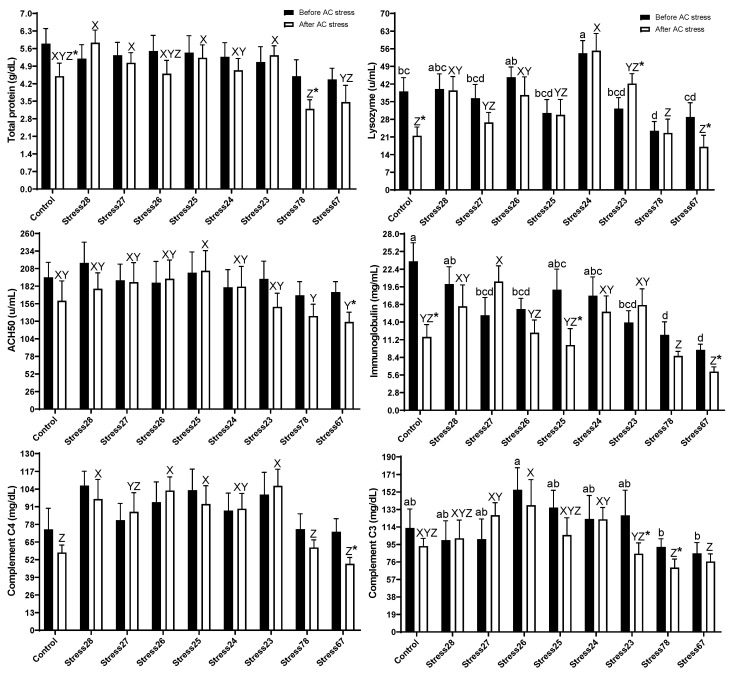
Immune response parameters of oscar were exposed to different schedule stress during ten weeks plus data related to after acute confinement stress (AC stress). Values were represented by means ± SDM of triplicate samples. Asterisk indicates the significant difference in each treatment before and after AC stress according to the independent sample *t*-test (*p* < 0.05). The letters a, b, c, and d; and X, Y, and Z indicated significant differences based on the Tukey test among groups before (nine treatments) and after AC stress (nine treatments), respectively.

In the present data, before stress, oscars treated with Stress78 (23.43 U/mL) had lower levels of lysozyme than Control (39.03 U/mL), Stress28 (40.04 U/mL), Stress26 (44.70 U/mL), and Stress24 (54.24 U/mL) (*p* < 0.05). Immunoglobulin followed the same trend, and Stress78 (11.96 mg/mL) and Stress67 (9.57 mg/mL) had lower contents than the Control (23.65 mg/mL), Stress28 (20.04 mg/mL), Stress25 (19.13 mg/mL), and Stress24 (18.20 mg/mL) groups. Further, Stress78 (92.24 mg/dL) and Stress67 (85.30 mg/dL) treatments had lower contents of complement C3 than the Stress26 (154.50 mg/dL) group (*p* < 0.05). After stress, Stress78 (3.20 g/dL) and Stress67 (3.46 g/dL) had lower contents of TP than Stress28 (5.85 g/dL), Stress27 (5.02 g/dL), Stress25 (5.23 g/dL), and Stress23 (5.34 g/dL) groups (*p* < 0.05). The ACH50 in Stress78 (137.80 U/mL) and Stress67 (128.90 U/mL) groups were lower than Stress25 (181.17 U/mL) treatment (*p* < 0.05). These two groups had the lowest content of immunoglobin, C4, and C3, as well (Figure 3). The AC stress also decreased immune parameters in those two groups compared to before stress (*p* < 0.05).

**Figure 4:**
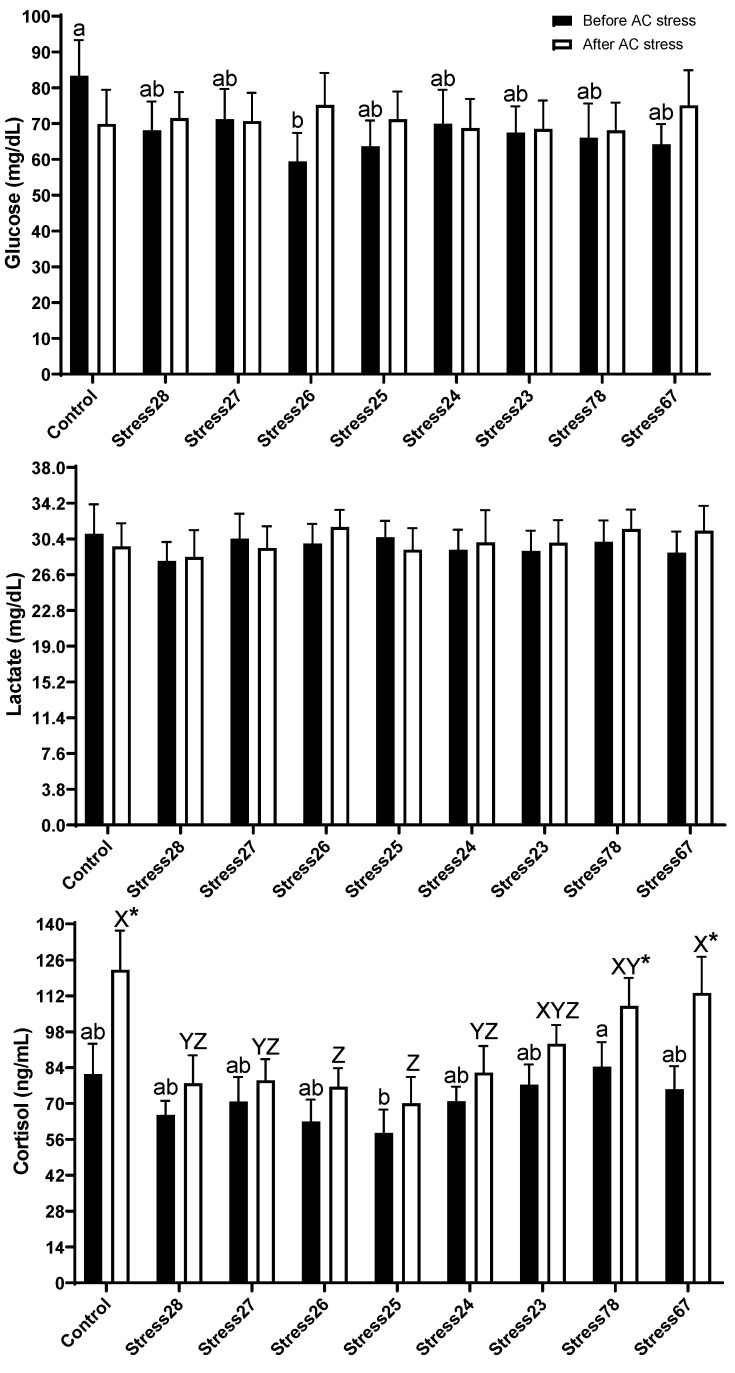
Stress response parameters of oscar exposed to different schedule stress during ten weeks plus data related to after acute confinement stress (AC stress). Values were represented by means ± SDM of triplicate samples. Asterisk indicates the significant difference in each treatment before and after AC stress according to the independent sample *t*-test (*p* < 0.05). The letters a, b and X, Y, and Z indicated significant differences based on the Tukey test among groups before (nine treatments) and after AC stress (nine treatments), respectively.

The present study indicated that there was no significant difference in lactate levels. Before stress, Control (83.33 mg/dL) had higher glucose levels than Stress26 (59.44 mg/dL) treatment. Cortisol in Stress78 (84.31 ng/mL) group was more elevated than fish exposed to Stress25 (58.50 ng/mL) schedule (Figure 4). After stress, the results showed the same trend, so that the Control (122.14 ng/mL), Stress78 (108.06 ng/mL), and Stress67 (113.13 ng/mL) groups had higher cortisol levels than Stress26 (76.53 ng/mL) and Stress25 (70.05 ng/mL) treatments. These three treatments had higher values after AC stress compared to before one as well (*p* < 0.05).

### 3.4. Antioxidant Enzymes Activities

**Figure 5:**
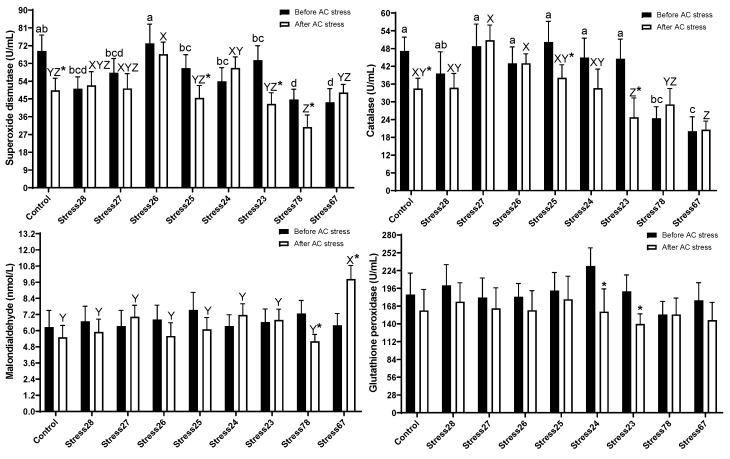
Antioxidant system parameters of oscar exposed to different schedule stress during ten weeks plus data related to after acute confinement stress (AC stress). Values were represented by means ± SDM of triplicate samples. Asterisk indicates the significant difference in each treatment before and after AC stress according to the independent sample *t*-test (*p* < 0.05). The letters a, b, c, and d; and X, Y, and Z indicated significant differences based on the Tukey test among groups before (nine treatments) and after AC stress (nine treatments), respectively.

At the end of the experiment, the current data before stress indicated that most antioxidant parameters were altered (Figure 5). Oscar exposed to Stress78 (44.46 U/mL, 24.47 U/mL) and Stress67 (43.30 U/mL, 20.04 U/mL) schedules had a lower value of SOD and catalase than the other groups. It can be seen that Stress78 had a lower value of antioxidant system enzymes, and this group was probably under stress (*p* < 0.05), as stress parameters were also shown. After stress, SOD in Stress26 (67.70 U/mL) group had higher content than Control (49.30 U/mL), Stress25 (45.46 U/mL), and consecutively stressed treatments (Stress23 (42.44 U/mL), Stress78 (30.78 U/mL), and Stress67 (48.25 U/mL)) (*p* < 0.05). Catalase showed the same trend, and consecutively, stress treatments had a lower value than Stress27 (50.83 U/mL) and Stress26 (43.00 U/mL) tanks. The Stress67 (9.87 U/mL) similarly had a higher value of MDA than other groups.

### 3.5. Liver Enzymes

**Figure 6:**
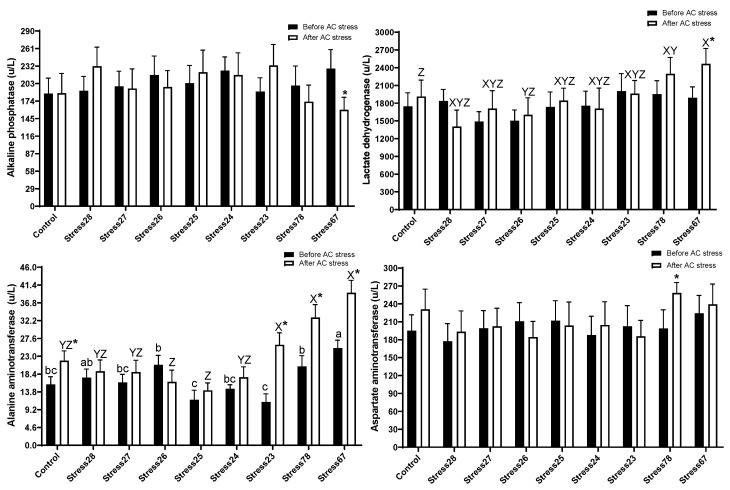
Liver enzyme parameters of oscar were exposed to different schedule stress during ten weeks plus data related to after acute confinement stress (AC stress). Values were represented by means ± SDM of triplicate samples. Asterisk indicates the significant difference in each treatment before and after AC stress according to the independent sample *t*-test (*p* < 0.05). The letters a, b, c and X, Y, and Z indicated significant differences based on Tukey Test among groups before (nine treatments) and after AC stress (nine treatments), respectively.

Figure 6 indicated there was no significant difference in ALP, LDH, and AST before stress. Stress26 (20.76 U/L), Stress78 (20.35 U/L), and Stress67 (25.12 U/L) groups had a higher value of ALT than fish that experienced Stress25 (11.76 U/L) and Stress23 (11.20 U/L) schedules (*p* < 0.05). After AC stress, Figure 6 showed a change in ALT and LDH levels. Stress78 (2295 U/L) and Stress67 (2466 U/L) groups had a higher value of LDH than Control (1913 U/L) (*p* < 0.05). The ALT in Stress23 (25.97 U/L), Stress78 (33.0 U/L), and Stress67 (39.4 U/L) groups was higher than the others (*p* < 0.05).

## 4. Discussion

The recovery time of fish from stress varied depending on the stress nature, severity, fish species, and experimental conditions. Recovery parameters of stress in fish have been cortisol, lactate, plasma osmolality, glucose, feeding behaviour, thyroxine, hematological indices [28], neurotransmitter enzyme, locomotor behaviour, and antioxidant enzymes [29]. In the present study, the effect of the time difference between EMS and AC stress (from one week to six weeks) and the consecutiveness of EMS on growth, immunity, body composition, antioxidant activities, blood indices, and liver enzymes were tested. This study was a follow-up study from our EMS project. After doing three studies [4,13,14], we understood that stress at two out of ten weeks did not impair growth, stress response, and most of the measured parameters, but it did improve the survival rate after AC stress. However, it raises the question of whether stressed fish in weeks 2 and 4 would respond differently than those exposed to stress in weeks 7 and 8. The result of this study indicated that the time of exposing fish to EMS is irrelevant. However, fish exposed for two consecutive stress weeks (7 and 8) could not be recovered after two weeks, and these groups had lower survival rates, immunity, and stress response. No study has compared the effect of EMS on measured parameters with the current schedule. More studies are required to test the impact of the consecutiveness of stress on other species.

### 4.1. Growth Performance and Body Composition

One of the most important impacts of stress in the long term is impaired growth. In the present research, two consecutive weeks of EMS, regardless of the occurred time (weeks 2 and 3, 6 and 7, or 7 and 8), impaired growth performance. It can be hypothesised that consecutive stress can play a key role in stress recovery. It seems that fish growth slowed down after weeks 7 and 8 by EMS, and oscar had no time to recover the occurred slowed growth as the experiment finished after nine weeks. Domesticated fish can somehow have better stress responsiveness than wild ones. However, it is suggested that more domesticated fish species, such as rainbow trout (*Oncorhynchus mykiss*) and carps, can have shorter recovery times. Domesticated perch larvae (*Perca fluviatilis*) coped better with exposure to thermal stress than non-domesticated animals [30]. Domestication modified stress responses in chickens regarding morphology, physiology, and behaviour [31]. Probably, oscar is not domesticated enough and behaves similarly to a wild fish.

Some fish species have a long-term response to different stressors. For example, it was observed that Chinook salmon (*Oncorhynchus tshawytscha*) did not feed for a long time (one month) after handling/sampling stress [32]. The time course for recovery of measured parameters (i.e., plasma cortisol, glucose, lactate, circulating lymphocytes, thyroxine) after stress was a minimum of two weeks for brown trout (*Salmo trutta*) [28]. In rainbow trout fish stressed daily (30 s in the air or draining water completely or chasing fish in the tank for 15 min), the growth was not different from that in controls after ten weeks [33]. However, daily, brief handling stress (10 s) over four weeks in farmed rainbow trout individually declined growth [34]. Some of these investigations are consistent with our data and highlight the important role of domestication (rainbow trout vs. Chinook salmon or brown trout), social communications, and the size of fish.

Differences in survival rates in the present research were insignificant among treatments, suggesting the culture condition was accepted by fish. Likely, EMS was not strong enough to affect the survival rate at the end of the study. The higher survival rate in EMS groups compared to Control is consistent with our previous studies [4,13,14]. The reason for the observed output in Stress78 can be frustrated from the recent EMS stress, and two weeks was not enough for the fish to recover from stress. However, this result was not observed in the Stress67 group, showing that this treatment could be recovered from the last EMS stress after three weeks. The treatments Stress28 and Stress27 did not differ in this parameter, even though the last EMS was less than a three-week distance from the final AC stress. The reason is that they experienced only one week of stress, and it was not severe enough to affect their abilities to tackle stress. As a result, in the current research, both recovery time and severity played key roles in the adaptability of fish to stress.

Internal factors, such as age, gender, and size, as well as external factors, such as water quality, season, and geographical location, all influence differences in the proximate body composition of aquatic species. However, diet is a major driver in changing body composition [35]. The lack of significant differences in proximate composition in our data somewhat aligns with earlier studies [4]. The lack of difference can prove that energy allocation did not change, and fish did not use fat or protein to provide energy to tackle stress [36].

### 4.2. Haematology and Blood Biochemistry

Environmental and nutritional stressors can drive changes in haematology and biochemistry parameters, and these parameters can be indicators of fish health status [37]. Similar to our previous EMS research, there was no significant difference in Ht, Hb, RBC, WBC, MCH, MCV, and MCHC among groups [4]. The reduced growth (while not significant) in Stress78 and Stress67 can be related to the lower content of BP in this group. Interestingly, in earlier studies from this project [4,13,14] and also other studies [17,26,38,39,40], a direct correlation between growth and BP was observed. After AC stress, oscar experienced the Stress67 (and Stress78 schedules had a lower value of BP than the other treatments (*p* < 0.05). These results could likely explain the lower survival rates in these groups, especially Stress78. Earlier studies indicated that a lower level of BP can indicate weaker fish in terms of health status. Similarly, when fish were fed too much soybean [39], carbohydrates [38], and meat and bone meal [17], their BP was found to be lower than the control group.

Stress can change the blood biochemistry, as it is an indicator of fish metabolism, and fish may use lipids and proteins in serum to provide energy to tackle stress [36]. Further, some blood biochemistry parameters, such as albumin and total protein, are also signs of the immune system status. In the current research, blood biochemistry was changed with stress schedules (Figure 2). Before AC stress, EMS groups had higher values of LDL than the Control. After AC stress, the same results for LDL value were observed so that Stress78 had a higher value than Control (*p* < 0.05). LDL cholesterol is often called “bad” cholesterol and is a sign of many health diseases in mammals. Stress78 group had lower survival rates, as well, and it can be connected to higher LDL levels. Results from our research also showed more elevated LDL levels in stressed fish [13,14]. Feeding high levels of soybean protein concentrate [41] and infected fish with *A. hydrophila* [42] showed the same results. Stress causes the body to produce more energy in the form of metabolic fuels, causing the liver to produce and secrete more LDL, the bad cholesterol. Furthermore, stress may impair the body’s ability to clear lipids [43]. Similarly, lipid components of blood (cholesterol, triglyceride, and HDL) were altered after AC stress [4], which is in line with our study in terms of LDL.

### 4.3. Immune and Stress Response

If stress exceeds the immune system’s normal adaptability, disease states or fatal conditions may occur [44]. Chronic stress also prevents immune cells and signalling networks from properly communicating [44]. Stress and the immune system can affect each other in different ways through the reverse path of immune function, ending with stress responses, particularly from the visceral system to brain function, behaviour, and stress coping [45]. In the present data, before stress, the decreasing immunity in Stress78 was observed, which in turn may have been responsible for the reduction in growth (while it was not significant). Some other negative signs from this treatment were observed in hematological parameters, as well. The AC stress also decreased immune parameters in Stress78 and Stress67 groups (*p* < 0.05). Stress78 and Stress67 groups were the worst treatments regarding the immune system after AC stress, and acute stress negatively affected these two groups more than others. These data can be matched with a lower survival rate in Stress27 fish. In our previous EMS studies also, the immune system in the Control group was lower than two-time stressed treatments [4,13,14]. This result shows that oscar was probably under stress due to two-week consecutive stresses, and the immune system was suppressed. However, this is a surprising result, as the stress was mild, and it only occurred twice per week for a total of four-times stress. We hypothesised that, even though these two weeks of stress did not affect measured parameters, this hypothesis was rejected. The Control group in the present data showed the same trend as our earlier EMS studies, and all these parameters were lower than other groups, but not the Stress78 and Stress67 fish. To the best of our knowledge, no study measures fish immunity under this kind of scheduled stress in animals. More research is needed to determine how different fish species’ immune systems react to stress and how this eventually affects growth performance.

Fish homoeostasis, particularly in the neural, endocrine, and immune system, can be negatively affected by stress. When stress is quick and acute, a stimulatory reaction occurs, while a prolonged response usually has an immune-suppressive effect in fish [46]. The severity of the stress, fish species, nutritional background, and the type of stress determines how much stress exposure harms fish physiology, growth, and survival rate. The present study showed that fish under treatment without consecutive stress were generally not harmed in terms of stress parameters, such as cortisol, glucose, and lactate. The first and second responses of fish to stress are cortisol, glucose, and lactate, respectively. The parameters can give extra information regarding the severity, duration, and recovery period of the stress response [47]. After AC stress, the Control, Stress78, and Stress67 groups had higher cortisol levels than Stress26 and Stress25 fish. These three treatments had higher values after AC stress compared to before one, as well (*p* < 0.05). It can be concluded that both consecutive stress and recovery time play key roles in cortisol levels and stress responsiveness. Control treatment also had a higher level of cortisol, which is in line with our previous EMS studies [4,13,14]. Collectively, these findings suggest that EMS can reduce the stress responsiveness of fish. The lower survival rates in Control and Stress78 groups can be linked to higher cortisol in these treatments. We did not have the facility and resources to test all possible scheduled tests, and more studies are required to understand how much time oscar needed to recover from two-week consecutive stress, and it seems that three weeks were not enough. It should be noted that different animal species require different recovery times and stress responsiveness, and testing this kind of schedule in other fish species is highly recommended. Fish can be under consecutive stress and, eventually, higher stress responsiveness, routinely, in aquaculture systems. Aquaculture sustainability highly depends on growth and survival rate, and reduced stress responsiveness can lead to an improvement in these parameters.

### 4.4. Antioxidant Enzymes Activities

Many parameters have been commonly measured to monitor fish health status under stressful situations. One of the most important ones is antioxidant enzymes, including SOD, CAT, GPx, and MDA molecules in fish. These enzymes play important roles in protecting cells from oxidative stress that cause superoxide and H_2_O_2_ radical damage [48]. At the end of the experiment, the current data before stress indicated that most antioxidant parameters were altered (Figure 5). It can be seen that Stress78 had a lower value of antioxidant system enzymes, and, probably, this group was under stress (*p* < 0.05), as stress parameters were shown, as well. The lower, but not significant, growth on this treatment probably can be connected to the lower content of antioxidant parameters. The link between growth and antioxidant activities was well reviewed [48], and a higher value of antioxidant activities can be linked to improved growth, health, and physiological condition. After AC stress, Stress78 fish in most of the parameters was the worst one. The lower survival rate in this group might indicate decreased cellular resistance to oxidative stress and impaired maintenance of the antioxidant-ROS balance. Other fish studies similarly showed decreased SOD and catalase levels with pollution stress [49] and ammonia exposure [50].

### 4.5. Liver Enzymes

Liver or serological enzymes, such as LDH, ALP, AST, and ALT, are frequently measured in fish physiology studies. Figure 6 indicated that Stress78 and Stress67 fish were under stress, and most liver enzymes were the highest in this treatment. Matching this data with the survival rate after AC stress and other physiological parameters mentioned in the last sections is further evidence that this treatment was not under “normal physiology”. Aquaculture studies have reported elevated ALT, AST, and decreased ALP in response to different stresses in various fish species [51,52,53,54,55]. Based on these observations, fish exposed to EMS schedules deal well with AC stress, resulting in no increase in liver enzymes. More research is necessary to demonstrate how stress and the liver’s physiological status react with each other.

## 5. Conclusions

The result of the present research suggests that both the time difference between EMS and AC stress and exposure to consecutive stress play key roles in survival rate and fish health. Stress78 fish that were exposed to consecutive EMS stress in weeks seven and eight had the lowest survival rate after AC stress, hematological parameters, immune response, antioxidant parameters, and liver enzymes. All of these alterations lead to impaired fish growth and survival rate after AC stress, even with two-week consecutive EMS. As this research has been a primary study, more programmed stresses and measurements of more parameters at the classical and molecular levels are needed to illustrate the various mechanisms of EMS in fish.

## Figures and Tables

**Table 1:** Experimental design for the effect of two-time scheduled stress (doing netting after the exchange of water on Monday and Friday of the proposed week) and AC stress at the end of the experimental period.

	Control	Stress28	Stress27	Stress26	Stress25	Stress24	Stress23	Stress78	Stress67
Week 1	Without stress	Without stress	Without stress	Without stress	Without stress	Without stress	Without stress	Without stress	Without stress
Week 2	Without stress	stress	stress	stress	stress	stress	stress	Without stress	Without stress
Week 3	Without stress	Without stress	Without stress	Without stress	Without stress	Without stress	stress	Without stress	Without stress
Week 4	Without stress	Without stress	Without stress	Without stress	Without stress	stress	Without stress	Without stress	Without stress
Week 5	Without stress	Without stress	Without stress	Without stress	stress	Without stress	Without stress	Without stress	Without stress
Week 6	Without stress	Without stress	Without stress	stress	Without stress	Without stress	Without stress	Without stress	stress
Week 7	Without stress	Without stress	stress	Without stress	Without stress	Without stress	Without stress	stress	stress
Week 8	Without stress	stress	Without stress	Without stress	Without stress	Without stress	Without stress	stress	Without stress
Week 9	Without stress	Without stress	Without stress	Without stress	Without stress	Without stress	Without stress	Without stress	Without stress
Calculation of weight of all fishes, growth, sampling of blood and other factors									
Week 10	Without stress	Without stress	Without stress	Without stress	Without stress	Without stress	Without stress	Without stress	Without stress
Final stress (AC stress) and sampling for serum and hematology parameters.									

**Table 2:** Growth performance of oscar (*Astronotus ocellatus*) is subjected to different schedules of stresses.

	Control	Stress28	Stress27	Stress26	Stress25	Stress24	Stress23	Stress78	Stress67
Initial weight (g)	5.57 ± 0.98	5.46 ± 0.68	6.27 ± 0.65	5.40 ± 1.05	5.60 ± 1.35	6.17 ± 0.67	5.40 ± 0.66	5.47 ± 0.67	5.80 ± 0.95
Final weight (g)	47.03 ± 7.16	46.96 ± 8.11	43.44 ± 8.95	43.53 ± 6.70	41.60 ± 7.37	44.86 ± 7.32	39.40 ± 4.62	32.23 ± 6.67	35.87 ± 6.21
Weight gain (g)	41.42 ± 6.26	41.50 ± 8.48	37.19 ± 8.61	38.13 ± 7.33	36.00 ± 8.16	38.70 ± 7.99	34.00 ± 4.30	26.78 ± 7.34	30.05 ± 7.16
SGR (%/day)	3.39 ± 0.11	3.41 ± 0.43	3.06 ± 0.26	3.32 ± 0.46	3.20 ± 0.60	3.14 ± 0.44	3.15 ± 0.16	2.80 ± 0.47	2.89 ± 0.54
FCR	1.89 ± 0.28	1.98 ± 0.31	2.09 ± 0.17	2.16 ± 0.19	2.25 ± 0.31	2.15 ± 0.37	2.30 ± 0.29	2.04 ± 0.34	2.09 ± 0.25
DFI (%/day)	4.72 ± 0.62	4.96 ± 0.66	4.93 ± 0.73	5.32 ± 0.54	5.36 ± 0.54	5.18 ± 1.22	5.52 ± 0.67	4.55 ± 0.95	4.76 ± 0.90
HSI (%)	2.33 + 0.31	2.66 + 0.31	2.45 + 0.28	2.42 + 0.22	2.43 ± 0.23	2.46 ± 0.19	2.42 ± 0.27	2.61 ± 0.34	2.46 ± 0.45
VSI (%)	5.87 ± 0.91	6.00 ± 0.62	6.23 ± 0.40	6.13 ± 0.38	6.10 ± 0.79	5.90 ± 0.80	6.10 ± 0.46	6.33 ± 0.45	5.90 ± 0.70
Condition factor	2.32 ± 0.46	2.59 ± 0.83	2.30 ± 0.59	2.31 ± 0.62	2.14 ± 0.72	2.37 ± 0.58	1.97 ± 0.35	1.85 ± 0.65	1.83 ± 0.15
Survival rate (%) ^#^	94.44 ± 6.36	94.44 ± 6.36	91.67 ± 4.17	93.06 ± 4.81	93.06 ± 6.36	91.67 ± 4.17	94.44 ± 6.36	91.67 ± 4.17	93.06 ± 6.36
Survival rate after AC (%)	60.00 ± 10.00 ^b^	80.00 ± 0.00 ^a^	83.33 ± 5.77 ^a^	76.67 ± 5.77 ^ab^	80.00 ± 10.00 ^a^	73.33 ± 15.28 ^ab^	73.33 ± 5.77 ^ab^	63.33 ± 11.55 ^b^	70.00 ± 5.77 ^ab^

Weight Gain = final weight − initial weight); SGR: Specific Growth Rate = ((Ln W2 – Ln W1)/63 days)) × 100; FCR: Feed Conversion Ratio = dry feed consumed (g)/weight gain (g); DFI: daily feed intake (%body weight.day^−1^) = 100× feed consumed (g)/((initial weigh+ final weight) × 0.5 × days); HSI: Hepatosomatic Index = (liver weight (g)/body weight (g)) × 100; VSI: Viscerosomatic Index = (visceral weight (g)/body weight (g)) × 100; CF: Condition Factor = (W^2^ (g)/Length^3^) × 100; ^#^ Survival Rate (%) = (number of fish in each group remaining at the end of experiment/initial number of fish: 20) × 100. The letters ^a, b^ indicated significant differences based on the Tukey test among groups.

**Table 3:** Carcass chemical composition (g/kg) of oscar (*Astronotus ocellatus*) under different schedules of stresses.

	Control	Stress28	Stress27	Stress26	Stress25	Stress24	Stress23	Stress78	Stress67
Protein	178.5 ± 15.7	176.9 ± 10.8	183.1 ± 10.1	180.2 ± 8.7	176.4 ± 9.5	179.3 ± 8.2	177.6 ± 4.4	171.8 ± 8.9	179.8 ± 8.7
Fat	59.1 ± 5.6	64.4 ± 8.7	60.7 ± 4.1	55.8 ± 4.9	60.5 ± 5.3	59.4 ± 4.3	62.3 ± 5.8	60.2 ± 4.2	60.6 ± 3.4
Ash	31.6 ± 2.5	28.6 ± 3.8	29.6 ± 2.5	29.3 ± 4.1	30.6 ± 4.0	31.6 ± 3.5	34.3 ± 2.1	31.0 ± 2.8	32.3 ± 3.8
Moisture	683.0 ± 23.2	674.1 ± 24.2	686.1 ± 29.7	679.3 ± 22.9	692.1 ± 38.6	673.3 ± 39.2	701.5 ± 21.8	699.9 ± 13.1	704.4 ± 17.4

Values are represented by means ± SDM of triplicate tanks.

## Data Availability

The data are not publicly available due to privacy or ethical restrictions.
